# Perceptions and experiences toward extended-release buprenorphine among persons leaving jail with opioid use disorders before and during COVID-19: an in-depth qualitative study

**DOI:** 10.1186/s13722-022-00288-4

**Published:** 2022-01-29

**Authors:** Anna Cheng, Ryan Badolato, Andrew Segoshi, Ryan McDonald, Mia Malone, Kumar Vasudevan, Beita Badiei, Allison Sugarman, Ross Macdonald, Jasdeep Mangat, Jonathan Giftos, Joshua D. Lee, Babak Tofighi

**Affiliations:** 1 Department of Population Health, New York University School of Medicine, New York, USA; 2 Division of General Internal Medicine and Clinical Innovation, New York University School of Medicine, New York, USA; 3 New York City Health + Hospitals, Correctional Health Services, New York, USA; 4 Albert Einstein College of Medicine, New York, USA

**Keywords:** Buprenorphine, Extended-release, Opioid use disorder, Depot injection, Criminal justice involvement

## Abstract

**Background:**

Extended-release buprenorphine (XRB) offers a novel approach to sustained monthly treatment for people who use opioids in criminal justice settings (CJS). This study explores the experiences of adults receiving XRB as a jail-to-community treatment.

**Methods and findings:**

In-depth qualitative interviews were conducted among adult participants with opioid use disorder (OUD; n  = 16) who were recently released from NYC jails and maintained on XRB after switching from daily sublingual buprenorphine (SLB). Interviews elaborated on the acceptability and barriers and facilitators of XRB treatment pre- and post-release. Interviews were audio recorded, transcribed, and analyzed for content related to factors influencing XRB treatment uptake and community reentry. Important themes were grouped into systems, medication, and patient-level factors. Key systems-level factors influencing initiation of XRB in jail included an alternative to perceived stigmatization and privacy concerns associated with daily in-jail SLB administration and less concerns with buprenorphine diversion. In-jail peer networks positively influenced participant adoption of XRB. XRB satisfaction was attributed to reduced in-jail clinic and medication administration visits, perceived efficacy and blockade effects upon the use of heroin/fentanyl following release, and averting the risk of criminal activities to fund opioid use. Barriers to retention included post-injection withdrawal symptoms and cravings attributed to perceived suboptimal medication dosing, injection site pain, and lack of in-jail provider information about the medication.

**Conclusion:**

Participants were generally favorable to XRB initiation in jail and retention post-release. Further studies are needed to address factors influencing access to XRB in criminal justice settings, including stigma, ensuring patient privacy following initiation on XRB, and patient-, provider-, and correctional staff education pertaining to XRB.

*Trial Registration* ClinicalTrials.gov Identified: NCT03604159.

## Introduction

Medications for opioid use disorder (MOUD; e.g., buprenorphine, methadone, Extended-release naltrexone) afford an effective approach to reducing the burdens and health harms of opioid use disorder (OUD) among CJS involved populations [1, 2]. While most people who use opioids (PWUOs) experience one or more incarceration events, provision of MOUD and SLB in CJS settings remains limited due to Medicaid suspension during incarceration, cost, security concerns, stigma, and insufficient corrections and clinical staff, and limited treatment knowledge among corrections staff and patients [3–7]. In a recent surveys among PWUOs with recent incarceration in MD and NY, ~30% reported receipt of non-prescribed SLB during incarceration, likely indicating a significant treatment gap [8].

While CJS populations with OUD are typically receptive to initiating SLB treatment during incarceration, [9] post-release treatment continuation remains challenging, with relatively high drop-out rates immediately post-release compared to new voluntary treatment episodes in community treatment [10, 11]. After release, patients often lack stable housing and employment, are re-exposed to drug-using networks, and commonly do not access community treatment resources due to lack of information, high costs, inactive Medicaid, and long wait times [12–14].

Buprenorphine extended-release (Sublocade, Indivior; XRB) offers clinicians and people who use opioids (PWUO) in CJS settings a novel approach to sustained monthly treatment while potentially reducing the risk of opioid reuse during the critical reentry period. This sustained-release monthly injection may also overcome other barriers to expanding access to MOUD in CJS settings by averting the risk of diversion and misuse associated with sublingual formulations of buprenorphine and affording more time to locate community treatment during reentry. Prior qualitative studies among PWUOs in non-CJS settings highlighted the benefits of XRB in reducing stigma, mitigating the risk of relapse following exposure to actively using peers, and avoiding contact with pharmacies [15, 16]. However, concerns regarding XRB among CJS patients during re-entry included medication safety and efficacy, preferences to self-initiate transitions between sublingual and extended-release formulations of buprenorphine, and cost [15].

Although XRB may overcome key obstacles to expanding access to SLB and MOUD in CJS settings, more data is needed to evaluate XRB patients’ experiences in jail and during re-entry. We conducted in-depth interviews among PWUO randomized to XRB in a recent NYC jail-based clinical trial to further describe treatment experience, treatment satisfaction, and barriers and facilitators to XRB initiation in jail and retention post-release. Interviews elaborated on nuanced elements of patient-centered care shaping the receipt of XRB, including transitioning between illicit opioids, methadone, and/or SLB to XRB, linkage to community treatment post-release, stigma, and the lived experience of receiving XRB during re-entry.

## Methods

### Study design

We conducted semi-structured, face-to-face, audiotaped interviews from October 2019 to May 2020 with 16 adults recently offered XRB prior to release from NYC jails. Participants were recruited from a parent randomized trial which was recruiting n  = 52 adults previously diagnosed with OUD and prescribed SLB by correctional health staff who were then randomized to XRB vs. continuation of standard daily sublingual buprenorphine prior to release, and followed for 8 weeks or longer post-release [17]. The New York University Grossman School of Medicine’s Institutional Review Board approved both the parent study and this qualitative protocol.

### Setting and population

SLB treatment for OUD is standard of care for adult detainees and sentenced inmates incarcerated in NYC jails. In the parent trial, recruitment, enrollment, randomization and treatment with XRB among a cohort of adult men and women already on SLB prior to a scheduled release date took place at two New York City Department of Corrections (NYC DOC) jail facilities within the Rikers Island jail complex. Upon release from jail, parent study follow-up visits were completed at Bellevue Hospital Center in Manhattan. Recruitment and visits in this qualitative study were usually scheduled alongside parent study visits at Bellevue.

### Recruitment

During the community follow-up period, the study team utilized convenience sampling to approach parent study participants randomized to XRB. Potential qualitative study participants were approached during scheduled follow-up visits or contacted by phone. Inclusion in the qualitative study was current enrollment in the parent trial, randomized to XRB, and released from jail. Inclusion in the parent trial included: (1) age over 18 years; (2) recorded diagnosis of OUD and currently on sublingual buprenorphine-naloxone; (3) incarcerated in NYC jail with upcoming scheduled release date; (4) willing to undergo randomization and potentially switch from SLB to XRB. Exclusion criteria included being unable to read or comprehend the informed consent. Persons that met the above inclusion criteria were approached at research visits and invited to participate in a brief and confidential interview. Study staff obtained informed consent from eligible participants.

### Data collection

After obtaining informed consent, the research staff (AC, RB, AS, MM, BT, JDL) conducted private face-to-face interviews lasting approximately 45 min. Participants were compensated with a transportation voucher and $50 USD following completion of the interview. Participants completed a demographic and clinical questionnaire. Medical students (RB, AS, BB) and research coordinators (AC, MM) trained in qualitative research methods conducted the interviews with the supervision of senior investigators (BT, JDL).

### Interviews

The open-ended interview script was based on recent and related studies [7, 13] and iteratively tested and refined by study staff (AC, MM, BT, JDL) during the initial completed interviews. Interviewers elicited and probed for nuanced explanations of perceptions and experiences relating to XRB, including factors influencing individuals to decline treatment pre/post-release, how knowledge or experiences with other MOUD (e.g., methadone, extended-release naltrexone, SLB) influenced receipt of XRB, and how XRB may have influenced decisions or experiences related to illicit substance use.

In response to the COVID-19 pandemic, most participants remaining on XRB as of March 2020 were transitioned back to SLB when Bellevue’s routine outpatient clinical operations halted and monthly injections were suddenly problematic. Additional interviews through May 2020 newly probed and assessed perceptions and experiences with XRB and OUD during COVID-19, both in the community and within NYC jails. The COVID-19 interview guide allowed the study team to elicit and probe for experiences related to XRB treatment with COVID-19 infection in jail, tapering off from XRB, and changes in illicit substance use within the community.

### Analysis

All interviews were audio recorded, transcribed, de-identified, and analyzed line-by-line by at least two independent coders [13]. Analysis and interpretation of interview findings occurred simultaneously by utilizing an iterative thematic coding process based on established qualitative research methods [18, 19]. Codes were developed after numerous readings of interviews and the grounded theory approach. Reviewers yielded key codes, sub-codes, and code ‘clusters’ that were organized into themes. The study team met weekly to discuss findings, develop and continuously refine the codebook after reaching consensus on emerging themes, and apply the resulting codebook to subsequent interviews. Intercoder reliability between the study team was ensured by individually coding transcripts and reviewing findings during weekly meetings. Participants’ responses regarding barriers and facilitators to XRB treatment and community reentry were organized into three areas: (1) medication-level, (2) patient-level, (3) systems-level, and (4) COVID-19-level factors. There was some coding overlap between these three levels. Key themes organized from the coded text included: patient experiences upon XRB induction, facilitators and barriers to XRB use pre- and post-reentry, changed peer interactions pre- and post-reentry, and facilitators to care upon reentry. Discrepancies and ambiguities pertaining to code findings were discussed with senior investigators until consensus was reached.

## Results

### Participant characteristics

The study team approached 17 patients who initiated XRB in jail for OUD under the pilot study, and 16 participants agreed to complete the individual interviews. Study participants were primarily male (n =13, 81.3%), with a mean age of 45 years (range 30, 55). Race/ethnicity were not re-collected among the 16 participants; the parent trial enrolled 81% Black, Hispanic, or self-reported ‘other’ adults. Respondents reported a prior history of injection drug use (n =7, 41.1%), alcohol use (n =5, 29.4%), and nonopioid illicit substances (n =10, 58.8%). In their lifetime, most participants were previously experienced with SLB (n =13, 76.5%) and methadone (n =12, 70.6%). Few had ever received extended-release naltrexone (n =3, 17.6%). The mean length of time from jail release to the interview date was 9 weeks (range, 3–16 weeks).

### Medication-related factors for initiation and retention on XRB

Most participants had no prior knowledge of XRB until approached by the study staff for enrollment in the trial during a jail incarceration and prior to a scheduled release date. Some participants compared XRB’s subjective effects to SLB with the benefit of experiencing its treatment effect for approximately 1 month:

*“They give it to you for a month—they give it to you once a month and it stays in your system… it’s like the equivalent between 2 and 3 strips [of SLB] a day. And it like just like coasts you through the month.” [43]*

Familiarity with daily SLB proved to be a motivating factor for participants to receive XRB. Nonetheless, some respondents expressed concerns about XRB, including precipitating fears of being exposed to needles during monthly injections and the reduced efficacy of a medication administered subcutaneously versus more reliable oral or sublingual dosing of MOUDs:

*“Alright so one of the issues was because I am definitely afraid of needles. I was apprehensive… the doctors explained to me it’s kind of like a TB shot it goes under the skin but it goes in the stomach so I was apprehensive but I did it and it wasn’t bad at all.” [46]*

*“You don’t know what’s the outcome or nothing, so it’s like you get the shot and you be like damn what’s going to happen, is my body going to reject to it, that’s the cue if you’re allergic to anything, but I was saying to myself if I was allergic to suboxone… then I definitely won’t get a reaction off of this because it’s almost the same thing. You know maybe it’s different because it’s going in your skin but other than that I ain’t have a problem with it.” [49]*

After receiving XRB, participant endorsement of injection-site pain consisted of soreness around the injection site, reports of bruising and discomfort adjusting to the palpable lump remaining in their subcutaneous abdominal tissue:

*“And then actually getting the shot was painful. It’s like a real, real bad stinging pain. You know, and after, you know, it’s like a lump. You know, with time it goes down. I’m guessing as the medication releases, it goes down. But it’s pretty annoying. Like when you are putting on your clothes, it’s painful. Anything that touches, it’s like you know, it bothers.*

*And then you get a real bad bruising around the area. That’s the only part, it’s bad.” [19]*

Some participants originally expressed trepidation about the medication’s efficacy in rapidly quelling cravings, pain, and withdrawal symptoms, but those concerns largely subsided after the resolution of those symptoms post-injection:

*“But when he took away the needle and the next day I went to work, I felt good, I didn’t feel pain… I feel pretty good. I’m still using it.” [06]*

*“After the shot, the first time, I felt like it wasn’t going to work… I gave it a couple of days, I knew I was going to get high again, was going to be buying the suboxone, but time passed through…two weeks, three weeks… and no getting high.” [06]*

Nearly all respondents reported no longer using illicit opioids while on XRB and some attributed their treatment success to having tested the blockade effect of XRB after using illicit opioids without experiencing any euphoria:

*“This time I was controlled. Like I said, you know, it’s really a block. You cannot get high no matter how much you try. No matter how much you do, it’s not gonna work. Your body rejects it...You can’t do too much on that at all. That’s actually a great thing, if you ask me.”[19]*

Participants commonly elicited a perceived sense of normalcy after initiation on XRB. Some attributed that feeling to the monthly treatment visits and pharmacological effects of the long-acting injectable that averted the onset of cravings and withdrawal symptoms that “reminded (me) of the addiction”:

*“...just knowing that you do it that one time and then I don’t have to worry about it all month. I don’t have to worry about if I lose my medicine, if I’m going to vacation or something. It’s just in you already, it’s like a thing to not worry about. Versus taking a pill, you gotta worry. Wake up each morning and make sure you have it. I just think the shot was better.” [21]*

*“You’re your regular self, you can go on every day and do whatever you want and you don’t have to be reminded of the addiction… I don’t have an addiction as far as I’m concerned...I’m not reminded every day that I’m taking something for the addiction because.. it’s not there... I don’t have to think about it at all.” [22]*

While XRB diminished opioid craving and heroin/fentanyl use as designed, stimulant use persisted for most.

*“The way I do heroin and cocaine, I do heroin and cocaine together, since eleven years old. And I leave heroin, I want to keep the cocaine. I’ll be honest. I want to keep the cocaine. I like the rush, I like the high, for the cocaine. This one works for the heroin, nothing works for the cocaine. It’s the same rush with the injection or without the injection.” [6]*

*“Yeah, I still have an issue using cocaine. And I really wish there was a shot for that. And you know what, I don’t even really want to do it but I still fucking do it. I just don’t understand myself, but I do it and I wish I didn’t but I do.” [43]*

### Patient-level barriers and facilitators

Participants highlighted a multitude of factors that facilitated their decision to pursue treatment with XRB while in jail. Patient-level factors included preferences for monthly injections on XRB in the jail OTP program, reduced risk of opioid reuse while incarcerated, and a desire to avoid opioid withdrawal symptoms and overdose episodes while incarcerated and post-release.

Most participants had been released from jail on either SLB or methadone in the past and expressed satisfaction with how their recent reentry experience on XRB prevented them from experiencing any withdrawal symptoms and reducing the potential risk of reengaging in criminal activities to procure illicit opioids:

*“A lot of people’s first thought it’s like, “Yeah I’m gonna get high.” When you come out on the shot it’s like “Nah.” I want some good food, you know, stay home, relax… It didn’t make me want to rip and run the streets. It’s like I’m good, I don’t feel sick. I don’t feel the need to have to go and have to get anything else.” [19]*

Participants frequently highlighted their improved likelihood of securing employment following reentry while on XRB since monthly depot injections and clinic visits were less likely to interfere with work schedules:

***“Yes and functionally I can work, I don’t wake up sick every day, don’t gotta make sure I got money to go to work to get drugs, seven in the morning so I could get up. I don’t gotta worry about that, so I’m able to do all these things.” [01]***

Three of the 16 interviewed participants transitioned from XRB to SLB within the 8-week study period. One participant decided to discontinue XRB due to their personal preference for the subjective “boost” that they experienced daily with SLB in place of the similar perceived effect they previously experienced with heroin and reducing their risk of engaging in illicit activities:

*“Especially in early recovery... complete abstinence is very difficult, for myself. I saw it as the lesser of two evils, in a sense... Meaning I would rather look forward to getting a little boost off of suboxone than sniffing heroin and having a habit and committing a crime to get it… It’s like taking alcohol away from an alcoholic. You’re going to be miserable for a little while.” [08]*

A second participant chose to discontinue XRB in light of significant post-injection site pain. The third participant felt persistent withdrawal symptoms and attributed to being under-dosed by clinicians following induction on XRB:

*“Maybe if I would’ve started at the correct amount I feel like I probably would’ve just stood on it. Like I said it’s more convenient. I wish they would find a way to work out that little stinging pain... As long as they find everybody’s level, I would advise it to anybody. I would personally do it again, myself, if it just...had a little fixing.” [19]*

### Systems-level barriers and facilitators

Participants highlighted a variety of factors that influenced their decision to initiate XRB, including their interactions with peers, CJS staff, and clinic staff. Prior to initiation of XRB, participants enrolled in the Key Extended Entry Program (KEEP), NYC’s jail opioid treatment program (OTP), described social pressures by other inmates to divert their daily doses of buprenorphine or methadone. Factors influencing diversion included financial need, social pressures within jail, and in one case, a desire to provide MOUD to those who were unable to enroll in the same OTP.

Financial concerns elicited by one participant forced them to reconcile between adhering to their prescribed dose of SLB or selling it for money to procure food. However, maintenance on XRB was perceived by another respondent to avert the risk of diversion regardless of any personal or social circumstances.

*“The thing with the pill is that you can take the pill. Like you wanna buy, and I don’t got food… I’m bringing it and giving it to you. With the shot, it’s different.” [33]*

*“If you ask me, that is what they need to prescribe in jail. Because anything else is making it out of there and it’s gonna be sold… If you really need that medication, you are gonna take your shot.” [19]*

Reduced interactions with corrections officers was elicited as a motivational factor for initiating XRB to daily MOUD as they were perceived to be stigmatizing or inattentive to patients’ health needs leading to more time-intensive processes to receive their daily doses of methadone or SLB:

*“It's that the staff [corrections officers] is just lollygagging. It’s the culture, that’s the word I’m looking for. The culture of the staff is a non-caring culture, they could care less about you. When they respond to an emergency they walk to an emergency. They don’t rush, they walk...” [46]*

*“I didn’t have to go every single day, you know. I wouldn’t have to get up and wait for escorts [corrections officers] and be locked in a cage waiting for hours for my medication.” [01]*

Privacy concerns were expressed by some participants with enrollment in SLB and methadone treatment services in jail. One respondent noted that attending a medical clinic daily revealed her status as a patient receiving MOUD, while a monthly visit for XRB was perceived by fellow inmates and corrections officers as a “*regular appointment, like the dentist, doctor, checkup, GYN [Gynecology]…*”* [19]*. Another participant recalled feeling “embarrassed” by the daily overhead announcements for them to arrive for their opioid medication visits:

“*In the Six Building, not everybody gets high. So you’re standing around with a bunch of dudes and there might be two or three of you. And when they call KEEP [the jail OTP] you have to get up and go and everybody knows your business. Everybody knows that [I’m] a dope fiend.” [24]*

Positive peer experiences with XRB encouraged some respondents to seek treatment with XRB. Participants acknowledged that hearing about the perceived clinical benefits of XRB from other prisoners maintained on the medication motivated their participation in the study:

*“Well in jail, word gets around…So when I was in the dorm, somebody was like, ‘Yo, they got this new stuff, this new study that they put in your skin and you don’t got to worry, they only got to do it once.’ I was like, ‘Alright, how you get to buy it?’” [49]*

*“I was trying to ask to receive as much information as I could from the fellow inmates that were in there that were receiving the shot. How did they feel and it made me feel like I could try because of the fact that they were explaining it to me and for them it helped. The ones that really want to try to stay off of drugs, it helps.” [30]*

One participant however recalled how XRB was still susceptible to the common critique of MOUD by misinformed friends and family as “substituting one drug for another”:

*“This friend of mine that I’m real close to, she’s like, ‘Don’t take that shot. What do you need that?’... Because to her it’s alternating a substance for another substance. But they don’t understand that it works man, it works. You know, what if it’s keeping me clean? [46]*

Although respondents typically learned about XRB from peers, they infrequently received information about XRB from their healthcare providers. One participant noted the difficulties of initiating treatment with a novel substance in jail, highlighting the limited information provided pertaining to its mechanism of action, risk of adverse events, and other pertinent details:

*“Sometimes, the nurse doesn’t explain anything to you. They’ll be like, ‘Yo, we got something new, this is the name. You wanna try it?’ and that’s it. But he’s supposed to explain to you everything, step by step, how it works, the side effects, everything. The nurse in the island don’t explain to you nothing. They give you the shot… ‘Do you want it, yes or no?’” [33]*

Challenges with long-term maintenance on XRB was elicited by some barriers and attributed to barriers to resuming XRB in community treatment due to limited availability and cost. One participant suggested initiating a peer support group specific to XRB recipients to collectively address concerns and share experiences pertaining to the medication:

*“I said I like talking with other people who have the shot. Hearing ideas, like what you’re going to do after this… when this is done. Are you planning on staying on this forever? What are you trying to do? How can you go about that, like what’s going to happen after this shot is off and then there’s no more?” [24]*

### COVID-19 pandemic: experiences and perceptions on XRB

Amidst the COVID-19 pandemic, participants described numerous challenges pertaining to OUD treatment and COVID risk. Most participants remaining on XRB were transitioned to SLB due to an outpatient clinic shutdown at Bellevue Hospital, NYC-wide stay-at-home orders, and the emerging success of buprenorphine telehealth visits.

This unexpected transition gave further insight into experiences transitioning from XRB to SLB. One participant noted that during his last scheduled monthly XRB injection, he was prescribed SLB. However, due to social distancing measures post COVID-19, he avoided going to the pharmacy to pick up his prescription of SLB and recalled not experiencing any cravings or withdrawal symptoms 5 weeks after his last injection:

*“So they got me script at the pharmacy, I have no way to pick that up yet. I’m getting ready to go and pick that up when we finish today…*y’*all told me it would last 4–5 weeks...I’m at 5 weeks. I ain’t had nothing else”. [41]*

Other participants expressed satisfaction with monthly injections since they could maintain social isolation if they were infected by COVID-19.

*“I believe that if I get sick I can stay home for fourteen days as long as I have my medication, my pill, my shot, I can stay in the house as long as I want”. [33]*

One participant highlighted XRB’s beneficial impact following the implementation of stay-at-home measures during COVID-19, including mitigating exposure to actively using peers or engaging in illicit activities:

“*You know. It’s not like the normal way of living. I see people just trying to get high, their regular habits. They not able to do that. Going crazy and stuff. Here I am, I don’t have to do that because I have something in my system, that I can wake up and not have to run to go do this and that.” [41]*

The narrative of a parole-supervised participant during re-entry, re-incarceration, and COVID-19 illness while on XRB highlighted both positive XRB treatment effects and the barriers presented by housing instability and parole conditions. The female participant was assigned to a women’s shelter by parole upon release from jail in Nov 2019, then found new private housing through a family friend and left the shelter, which resulted in a new technical parole violation for unauthorized change of address and re-incarceration in NYC jail Feb–April of 2020. She then contracted COVID-19, was isolated in a COVID-19 housing unit, and was then abruptly released prior to the scheduled release date. She then resumed XRB at Bellevue Hospital.

## Discussion

Findings from this qualitative study among CJS participants with OUD initiating XRB underscore the various medication, patient, and systems-level factors that facilitate and bar XRB initiation and retention. To our knowledge, this represents initial and novel qualitative data reinforcing the potential importance of XRB in improving the OUD corrections-to-community treatment model.

Despite initial apprehension about starting a new and unfamiliar buprenorphine formulation while incarcerated, most people tolerated the medication well and cited advantages of XRB over SLB treatment, including the enhanced sense of “normalcy” by avoiding unwanted contact with correctional staff for daily SLB administration and peers pressuring patients for diverted SLB, and during reentry when monthly clinic visits for XRB injections were more convenient to daily or weekly clinic visits associated with SLB or methadone for securing employment. Our findings are consistent with previous findings of the acceptability of long-acting naltrexone in CJS populations, [1, 13] and desirability of XRB initiation versus SLB among non-CJS OUD populations [15, 20].

Four participants admitted to using heroin to test XRB’s partial receptor agonist effect despite being informed of its mechanism of action by the study team. Nonetheless, these participants continued XRB injections as planned, citing their satisfaction in XRB’s potent blockade effect. This supports XRB’s characteristic blockade as favorable among users and is consistent with patient behavior on other forms of monthly OUD medication [10]. Urine results from the parent trial indicated most non-prescribed opioid use among all participants was mixed heroin-fentanyl, indicating the widespread availability and cross-contamination of both types of opioids in NYC during 2019–2020 [17].

The few participants who discontinued XRB after their initial injection cited the subjective desire to experience the daily “boost” of SLB, while others were unable to tolerate injection site pain and persistent cravings or withdrawal symptoms attributed to insufficient dosing of the medication. Given the dosing schedules available for XRB (100 mg or 300 mg), prescribers of XRB should stay in close contact with patients recently initiated on the medication to ensure proper dosing and prevent withdrawal symptoms and potential opioid reuse.

Reentry is an especially worrisome period for patients, as current research suggests there is an increase in overdose death. Reasons for this are multifactorial, including reduced opioid tolerance, limited access or inadequate linkages to addiction treatment services post-incarceration, insufficient social supports, housing instability, and employment opportunities upon reentry that further exacerbate the risk of opioid reuse and overdose [21–23]. Findings from our study described how XRB can partially offset some of these barriers given the convenience of monthly clinic visits for XRB injections versus likely more frequent clinic visit intervals for patients maintained on SLB or methadone that may interfere with employment and pro-social behaviors.

Our findings not only confirm persistent barriers to MOUD adoption upon re-entry, but also highlight the need for increased health education and peer support networks to link with addiction treatment, harm reduction, and social services as they transition back to the community. All participants interviewed reported lack of prior knowledge of XRB and access to information on XRB in jail, yet high favorability to initiating XRB if they were informed by health staff about XRB. This finding underscores the novelty of the therapy, with study participants drawn from the first large implementation study XRB in a US urban jail system (to our knowledge). In addition to routine counseling and education on XRB and other MOUD by jail clinical staff, findings from our study also highlight the importance of in-jail peer networks as an influential source of health information dissemination pertaining to XRB [12].

Participants felt that XRB positively impacted their interactions with peers and CJS staff as well as their quality of life during and post-incarceration. XRB was perceived to effectively eliminate social pressures to divert one’s daily doses of SLB or methadone, or misuse SLB. XRB treatment also has important systems-level implications for OUD treatment during- and following the COVID-19 pandemic. Within the jail setting, XRB treatment mitigates potential COVID-19 exposure by eliminating daily contact with corrections and healthcare staff delivering SLB or methadone to individual units or exposure to peers in the event of diversion. Within the community setting, XRB treatment decreases the need to commute to in-person clinic visits and pharmacies to receive prescribed doses of SLB or methadone and averting exposure to COVID-19.

Limitations to this study include the small sample size (n =16), participant demographic and substance use characteristics limited mostly to heroin use that may not be representative of the general population of PWUOs detained in jail, participant recruitment from a single jail setting, and selection bias due to interviews conducted with respondents still engaged in study follow-up visits that may not reflect sentiment and experiences among participants lost-to-follow-up. Further studies are needed to ascertain participants’ long-term experiences with XRB beyond the first 2 months of treatment. Lastly, since participants were interviewed during the first two months of XRB treatment and administered 300 mg injections, additional studies are needed to assess patient experiences following receipt of lower doses of XRB (100 mg) in the 3rd month of treatment and after discontinuing treatment entirely.

## Conclusion

XRB treatment during jail-to-community reentry was perceived by participants as a favorable treatment approach to reduce their risk of opioid reuse, illicit activities, stigma associated with daily receipt of MOUD, and COVID-19 exposure. Common reasons for discontinuation of XRB included post-injection site pain, desires to feel the subjective daily euphoric effects SLB, and perceived insufficient dosing of XRB. Further efforts are needed to educate patients, providers, and correctional staff regarding XRB and explore the role of peers and peer support groups to facilitate patient initiation on XRB.

## Data Availability

Not applicable.
